# Challenges experienced by health care professionals working in resource-poor intensive care settings in the Limpopo province of South Africa

**DOI:** 10.4102/curationis.v42i1.1921

**Published:** 2019-03-26

**Authors:** Hulisani Malelelo-Ndou, Dorah U. Ramathuba, Khathutshelo G. Netshisaulu

**Affiliations:** 1 Department of Advanced Nursing Science, University of Venda, Thohoyandou, South Africa

## Abstract

**Background:**

Providing optimal care to critically ill patients poses challenges in resource-poor settings because of the lack of equipment, inadequately trained personnel and limited infrastructure.

**Objectives:**

This study explored challenges experienced by health care professionals working in resource-poor intensive care units.

**Method:**

A qualitative, explorative, descriptive design was used. The population comprised nurses and doctors working in an intensive care unit of one hospital in the Limpopo province of South Africa. A purposive sample was selected and 17 semi-structured interviews were conducted. Data were analysed using Tesch’s method. Ethical considerations were adhered to.

**Results:**

Participants experienced challenges related to provision of suboptimal patient care, the challenge of non-adherence to protocols and/or instructions and the challenge of practising beyond the scope of practice.

**Conclusion:**

Lack of resources resulted in providing suboptimal intensive patient care. Patients were prone to infections and their safety might be compromised.

**Keywords:**

health care professionals; intensive care units; critically ill patients; resource-poor intensive care units.

## Introduction

Proper management of critically ill patients requires adequate material, human, infrastructure and financial resources (Kwizera, Dünser & Nakibuuka 2012). Worldwide, there is a problem in health care services regarding resource allocation. However, in intensive care units (ICUs), this problem is exacerbated by the fact that patients are critically ill and require expensive treatments, life support machines, skilled personnel and constant monitoring (Riviello et al. 2011). Monitoring of ICU patients has high financial implications, including equipment and consumable costs, training nurses and their salaries and the recruitment of specialist doctors (Murthy, Leligdowics & Adhikari 2015).

Every health care system has to cater for critically ill patients, regardless of the availability of resources. However, in many low-income countries, ICUs are underdeveloped because they require expensive resources (Murthy et al. 2015). Even in high-income countries, physicians have to make decisions regarding resource allocations in ICUs (Christian et al. 2013). Although there is an increase in the number of ICUs, access remains limited to these facilities. Despite the availability of ICUs, many patients die because of the lack of material and trained human resources to provide due care (Haniffa et al. 2017). Shortage of resources is sometimes related to increased demands for health care services, linked to population growth and changes in demographic profiles. An increased number of illnesses such as renal failure, diabetes mellitus and cardiac disease require critical care. As the population continues to grow, the shortage of critical care services for the given population becomes more apparent (Soltani et al. 2015). Every ICU has an inherent ‘capacity’ or ‘ability to provide high-quality care for everyone who is or could become a patient in that ICU on a given day’. However, there might be a need for resources that are not available and/or inadequate to provide the desired standard of care (Kohn, Halpern & Kerlin 2016).

In sub-Saharan African (SSA) countries, the high level of critical illnesses is related to the increased burden of HIV and AIDS, malaria and trauma, and to manage these critically ill patients, resources are needed (Kwizera et al. 2012). This burden is expected to increase with increasing population and increased access to hospitals (Murthy et al. 2015). In order to curb the demand and supply gap, changes concerning admission and referral practices and/or increased numbers of allocated ICU beds and skilled health care professionals are required. If the number of beds is not increased, not all deserving patients will be able to access ICU care. ICUs in tertiary and specialist hospitals are better equipped than those in the rural public hospitals. Private hospitals’ ICUs are only accessible to persons with medical insurance. Only 5% of the population benefits from the poor-resource ICU settings that are struggling to provide optimal quality care because of numerous challenges health workers face daily.

Limpopo province is predominantly rural and has an estimated population of 5.5 million people. The province has one tertiary hospital in Polokwane and is located 150 km from Vhembe district where the study is undertaken. Vhembe district has seven hospitals and one mental health institution.

The shortage of human resources is a major issue in Limpopo, and posts are not filled because posts have been frozen for some time. While many doctors and nurses remain unemployed, there are not enough open positions to employ them and enrolled nurses who furthered their studies privately are still practising as enrolled nurses as there are no posts for registered nurses. Intensive nursing is a speciality and requires further training in a high care setting and only a few registered professional nurses have been trained and some resign after training for greener pastures. The problem was also highlighted by the Minister of Health in Limpopo that as a rural province it is a struggle to attract specialists and senior doctors (Budget Speech 2017). According to the National Department of Health, there are currently over 44 000 funded posts in Limpopo. Of these only 35 450 are filled, leaving almost 10 000 vacant posts in the province. This translates into almost 25% of the workforce. A Human Sciences Research Council (HSRC) report showed the vacancy rate of doctors at 75% and nurses at 67% (Media statement – The Treatment Action Campaign 2017). Ntuli and Maboya (2017) indicated that the doctor–population ratio for Limpopo province in 2015 was 16.4/100 000 and Vhembe had 113 doctors and one specialist at a regional hospital. Furthermore, Ntuli and Maboya (2017) reported that health professionals in Nigeria, Ghana and Peru are more in favour of urban setting irrespective of measures such as compulsory community service and rural allowance. St-Pierre, Anderso and Saint-Jean (2011) also reiterated that Quebec too has been affected by the shortage of staff, particularly in the area of critical care: 50% of the nurses who practise in critical care have less than 5 years’ experience and as such nursing in ICU is becoming ever more complex and more intense, and nurses cannot quickly acquire the specific skills they need to practise in ICU without the support of experienced peers.

Health facilities infrastructure in Limpopo is often aged and run down, dysfunctional or inappropriate to the needs of patients. Most of the facilities were built by the missionaries and are in a dilapidated state. The ICU at the regional hospital has only four beds and a side room and caters for six district hospitals and the majority of the population without medical aids rely on its service that is not functional. The South African Medical Association (SAMA) in the province raised concerns that the procurement of basic equipment (i.e. casualty ventilators, arterial blood gas machines, anaesthetic machines and many others) moves at a slow pace which leads to rising morbidity and mortality from preventable conditions in regional hospitals. In addition, regional hospitals continue to exist without functional high care units. Challenges of resources in the province place health professionals under stress.

## Purpose of the study

The study sought to explore and describe the challenges experienced by health care professionals working in resource-poor ICUs.

## Research question

The main question was the following: ‘what are the challenges that you experience as a health care professional working in resource-poor ICU?’

## Research method and design

### Study design

The study adopted a qualitative, descriptive and exploratory design to answer the research question and achieve the objectives of the study by obtaining accurate information about the challenges health professionals experience while working in a resource-poor setting in a selected regional hospital in the Vhembe district of the Limpopo province. The exploratory design was used because little was known about the challenges health care professionals experience while working in resource-poor ICUs (Brink, Van der Walt & Van Rensburg 2012).

### Population and sample

The population comprised trained professional nurses and doctors working in ICUs. A non-probability purposive sampling was used to select intensive trained professional nurses and medical doctors who were consulting patients within a period of 1 year in the ICUs. A sample size of 22 participants was chosen as the best suitable sample, which had experience of working in ICUs and volunteered to participate. Data saturation occurred with 17 participants: seven doctors and 10 ICU-trained professional nurses. The participants’ ages ranged from 35 to 56 years, with seven of the professional nurses having more than 10 years’ experience and three had less than 10 years’ experience. Four medical doctors had 5 years’ post-internship experience and only three had less than 5 years’ work experience.

### Data collection

In-depth individual interviews were conducted with ICU-trained professional nurses and medical doctors sharing the challenges they experience while working in the ICUs. Interviews were conducted separately at separate locations determined by the participants in order to allow them to speak freely while the researcher noted what was discussed and audio-recorded the interview sessions. The central question was the following: ‘what are the challenges that you experience as a health care professional working in poor-resource ICU?’ Subsequent probing questions followed to encourage the participants to elaborate and clarify, such as ‘are the challenges related to patient care; resources or any other related to promoting or obstructing your functioning in the ICU?’ The interview was conducted in English even though a few participants used local language to emphasise their point and direct translation was done by the researcher.

### Data analysis

Tesch’s open coding method of data analysis (Creswell 2014) was used in developing themes and sub-themes and direct quotes were extracted from the transcripts. Field notes were also analysed so as to strengthen the direct quotes from transcripts and develop relationships. Data analysis was performed simultaneously with and after data collection from April to July 2017, assisting the researcher to refine the questions and to remember what was said or observed. The interviews were transcribed verbatim and studied using codes. The transcriptions were read and re-read to enable the researcher to become immersed in the data and sensitised to important issues. The data were analysed and coded by the researcher.

### Measures to ensure trustworthiness

Researchers adopt various strategies to ensure trust-worthi-ness of data as suggested by Lincoln and Guba (1985). Credibility was ensured by spending more time with the participants to establish rapport at their homes and at doctors’ residence within the hospital and to explain the aim of the study. The researcher spent at least an hour gathering data. Data were collected until data saturation had occurred as the researcher had to revisit the participants to get clarity and for participants to verify the transcript. The interviews were conducted in English mixed with the local ethnic language used in the region to enhance greater self-expression. The audio recorder, observational notes and verbatim transcribed interviews enhanced the truthfulness of the data.

To ensure transferability, the researcher selected information-rich participants through purposive sampling. The researcher provided dense descriptions of the research data, including verbatim quotations. Dependability was achieved by allowing cross-checking of codes also known as inter-coder agreement by the supervisor and the colleague in the Department of Nursing to see whether the experts would code the data in the same way as the researcher had performed. Throughout the study, data checking was performed and sustained discussions between researcher and supervisors enhanced the truth value of the data.

### Ethical considerations

Permission to conduct the study was sought from the management of the hospital after getting approval from the Limpopo province (SHS/PDC/32/3008). Permission to collect data was also sought from the operational manager of the intensive care unit at the regional hospital. Appointments were made with the participants before the date of data collection. Ethical consideration was followed to enhance protection of the participants, such as informed consent, and explanation was given about the study purpose and thereafter written consent was acquired. The participants were informed that field notes would be written and that an audio recorder would be used during interviews to capture the proceedings of the interview sessions. The participants were assured that any information provided would not be published or reported in any manner that would reveal their identity, and no information would be shared with unauthorised persons. The participants’ right to self-determination was also respected as participants were not forced to participate in the study and they were free to withdraw from the study anytime they felt uncomfortable.

## Findings

### Demographic data

Seventeen participants consisting of seven doctors and 10 nurses comprised the sample for the current study. Seven professional nurses had more than 10 years of experience as ICU nurses and only three had less than 10 years’ experience. Five of the nurses had postgraduate qualifications. Out of the seven doctors, four had 4 years’ experience post-internship and only three had less than 5 years’ experience.

Six sub-categories emerged during data analysis (Table 1). They reflected the challenges experienced by nurses and doctors working in a resource-poor ICU. Participants were identified by numbers and codes (D for doctor and N for nurse).

**TABLE 1:** Themes, categories and sub-categories of the study.

Themes	Categories	Sub-categories
Participants experienced challenges related to lack of or insufficient resources	Lack or shortage of material resources	Limited ICU beds Shortage of life-saving equipment Shortage of consumable supplies
	Lack or shortage of human resources	No intensivist Inadequate ICU-trained staff Practising beyond the scope of practice

ICU, intensive care unit.

### Limited intensive care unit beds

Limited ICU beds create a challenge for participants. Doctors expressed challenges related to not finding a bed when they wanted to admit a patient to ICU. Nurses’ challenges were related to their duty of saving patients’ lives. These sentiments are aptly expressed in the following excerpts:

‘One of the main challenge is created by the fact that this ICU only has four beds, therefore, that means these beds are not enough for the patients of Vhembe District. And it means that there will be times when I need a bed in ICU and ICU is full.’ (Participant 17, D, male, 38 years old)

‘Quite often we are faced with the situation where we see a patient and we need to decide, based not only on the condition of the patient but on the availability of ICU beds, whether we are going to take this patient to ICU or not. This is an ethical dilemma more than a medical dilemma because medically speaking you`ve got no doubt that this patient needs ICU.’ (Participant 11, D, male, 40 years old)

The study revealed that not all patients who deserved to be admitted to ICUs could be admitted because of limited beds because admission to an ICU was not only based on the condition of the patient but also on the availability of beds. Furthermore, another participant indicated that:

‘Some of the lives that are lost could have been saved if there were enough ICU beds as they have to play God by choosing the patient who has the chance for survival so that he can be the one taken to ICU if there are more patients than the available beds in ICU.’ (Participant 3, N, female, 45 years old)

Intensive care unit beds are a scarce resource and balancing the need for ICU beds with the available beds remains problematic.

### Shortage of life-saving equipment

Participants indicated that shortages of ventilators in the ICU posed challenges because it was impossible to provide mechanical ventilation to needy patients causing some patients’ deaths because they could not be ventilated. Participants also indicated that patients transferred to a tertiary hospital might not be accepted if the reason for such a transfer was the lack of ventilators. A medical practitioner had this to say:

‘I remember there was one patient who died because of near drowning, he needed a ventilator, there was no ventilator, so that patient died.’ (Participant 13, D, female, 40 years old)

Similarly, a nurse reiterated:

‘A well-functioning ventilator has some complication and what more if this ventilator is not functioning well. We are putting these patients in danger. Seated as we are, I cannot show any ventilator, which is functioning properly, which I can say if anything wrong happens to the patient it was just an accident. This is the biggest challenge because I have to help the patient anyway.’ (Participant 1, N, female, 55 years old)

Participants further indicated that:

‘there are not enough ventilators for all patients that can be admitted in ICU, there must be four ventilators for four patients and the hospital has only one reliable ventilator.’ (Participant 14, N, female, 53 years old)

‘Moreover some of the patients even die because of lack of ventilators and malfunctioning ventilators are used for the patient which is risky. The ventilators have not been serviced for long and sometimes patients are being subjected to improper management as they are kept on T-piece instead of ventilator.’ (Participant 3, N, female, 45 years old)

One participant indicated that:

‘there is one blood gas machine in the hospital, which is located in ICU. This blood gas machine can spend more than 6 months not functioning.’ (Participant 7, N, female, 48 years old)

Other participants stated:

‘Sometimes you will find that the monitor that the patient is using does not show saturation. In order for you to monitor the patient’s saturation patients have to share the oximeter.’ (Participant 4, N, female, 56 years old)

‘Only one cardiac monitor has all the parameters.’ (Participant 1, N, female, 35 years old)

‘The worst is to ventilate the patient without the blood gas machine.’ (Participant 7, N, female, 48 years old)

### Shortages of consumable supplies

The ICU did not have consumable materials and medicines. The ICU seemed not to be getting some items just because it is a regional hospital. Some of the items can only be found in tertiary hospitals. Participants indicated that patient care is compromised as they are risking patients’ lives. The concerned health professionals said:

‘I find it very strange that to get Panado for intravenous use, I must first write a motivation. This is the only treatment for pyrexia that I could give to our patients but you have to write a lot of papers and it will take days before you get it. Patients here are like patients in Polokwane.’ (Participant 11, D, male, 40 years old)

‘More than being a dilemma I think it is a political problem, because we are regarded as people who are in the rural, and we usually do not get equipment or whatever item that is used in ICU. We are categorised as people who are not supposed to get certain things.’ (Participant 2, N, female, 35 years old)

Participants revealed that some of the prescribed treatments took 2–3 days before they were administered as the pharmacist had to get them from other hospitals. This practice had negative impacts on the prognosis of patients as the first few hours are vital to the outcomes of the patient. Participants reported:

‘Sometimes we are forced to use the same suction catheter for the whole day because they are out of stock.’ (Participant 4, N, female, 56 years old)

‘We sometimes spend a lot of time without suctioning catheters size 14 or 12 and are the ones that are user-friendly to patients and then we tend to use one in all the orifices, the nose, the trachea and the mouth. So our patients are exposed to infections of the lungs.’ (Participant 15, N, female, 37 years old)

‘Because items are always out of stock, when they are available we order them in large numbers.’ (Participant 8, N, female, 44 years old)

### No intensivist

An intensivist is a certified critical care physician who provides clinical care exclusively in ICUs. Participants indicated that nurses took most of the decisions and this could lead to mismanagement of patients. Patients were reviewed once a day but sometimes (especially over weekends) they were not reviewed at all, because no intensivist was available:

‘ICU patients are reviewed once a day because there is no ICU doctor. Doctors sometimes come to ICU after finishing their ward rounds, which might be late in the afternoon. These create a challenge for nurses because until the doctor comes, there will be no new prescription.’ (Participant 5, D, male, 42 years old)

Professional nurses mostly managed patients when they could not reach the physician on call and this could result in medical errors. Doctors had to endorse something that had been administered by nurses in their absence, even if they felt it was not the ideal treatment:

‘Sometimes I don’t know whether to give treatment or not, what if the doctor does not prescribe the same medication. These usually create problems when I have to answer why I gave that medication.’ (Participant 15, N, female, 37 years old)

Nurses in the study did not receive the necessary support from the doctors although doctors relied on nurses for making some treatment decisions:

‘Maybe if ICU was to be looked after by someone like an anaesthetist who is well conversant with these machines. So the only thing that saves this ICU is the fact that the nurses know these machines. I think there was supposed to be a trained doctor working there because ideally, doctors must support the nurses but in our case nurses support the doctors.’ (Participant 17, D, male 38 years old)

‘Nurses are supposed to be given orders by a doctor but in our case, it is vice versa. Instead of helping us, we end up helping them. The nurses take most of the decisions and, the doctors just endorse.’ (Participant 3, N, female, 45 years old)

### Inadequate intensive care unit–trained staff

Intensive care unit patients require specialised care and specially trained staff to provide quality care. Participants highlighted that patient care was compromised because of inadequate numbers of trained staff by stating:

‘The allocation of our ICU leaves us with no choice but to use lower categories of nurses like staff nurses to nurse critically ill patients. It is a dilemma because I don’t have other staff, I only have those.’ (Participant 4, N, female 56 years old)

‘ICU only have twelve trained personnel that are not enough to provide quality nursing care. Sometimes the registered enrolled nurses and the inexperienced registered nurses are allocated to nurse ventilated patients, with the aim that they will be supervised. The supervision is always inadequate because whenever there is a very ill patient, the trained registered nurse, who is supposed to be supervising the untrained nurses, will be caring for the very ill.’ (Participant 14, N, female, 53 years old)

Participants had to work more than the required months of night duty because of staff shortages. Shortages of trained nurses resulted in delays in carrying out doctors’ orders. Some doctors just wrote their orders on the patients’ charts and such prescriptions were noticed during report giving. Some participants indicated that:

‘Sometimes you find that you are the only trained nurse on duty and you have to run around like a headless chicken. Someone will be calling you this side and the other one will be calling you that side. This is tiring; sometimes you miss some important things. You can also make some wrong decisions.’ (Participant 1, N, female, 55 years old)

‘Because there are few trained nurses, sometimes we have to go for night duty for more than 5 months. This is tiring.’ (Participant 10, N, male, 36 years old)

### Practising beyond the scope of practice

Nurses are faced with situations where they had to act beyond their scope of practice to save patients’ lives. Patients were resuscitated without a doctor. Doctors faced dilemmas when they had to decide whether or not to endorse something that had been initiated by nurses in their absence. Participants expressed their sentiments as follows:

‘For that moment, I have to think beyond my scope and do what was supposed to be done by a doctor. For example if I find that the patient is no longer breathing, I have to intubate the patient.’ (Participant 2, N, female 35 years old)

‘During emergency, this dilemma will hit back to ICU sisters also. They will have to assume the responsibility while the doctor is coming or is somewhere that is not the ideal ICU.’ (Participant 11, D, male, 40 years old)

‘There are changes that must be implemented immediately to the patient. For example, changing prescribed IV fluids to the patient because of certain results. It might be lab results or our blood gas machine. Most of the things I am saying are a bit beyond my scope but needs me to make some changes while waiting for the doctor.’ (Participant 12, N, female, 42 years old)

## Discussions

Working in resource-poor settings comes with many challenges, but the ICU poses more detrimental challenges because it has to deal with the lives of patients and providing quality of care to critically ill patients. Vhembe district of Limpopo province is experiencing challenges in provision of intensive care. Participants indicated that the number of ICU beds in this hospital is not compatible with the number of people it serves. Vhembe has a population of 1 393 949 and has six district hospitals with a limited capacity of ICU beds. The limited number of ICU beds at the provincial hospital also makes it impossible to transfer patients for further management because of limited ICU beds. Doctors and nurses had challenges of admitting patients in the ICU because every deserving patient should be given a chance of being admitted to an ICU. The greatest challenge, caused by the limited number of ICU beds, was being forced to make a choice on which patients deserved the ICU bed most. Participants emphasised that the shortage of ICU beds delayed some patients’ admissions to the ICU, resulting in complications. The results of this study are supported by Naidoo, Singh and Lalloo (2013) that, worldwide, the demand for ICU beds exceeds the supply causing daily shortages of ICU beds. In South Africa, unfair distribution of resources is among the challenges that the doctors and nurses face because of unequal distribution of resources between the private sector and public sector; again, the public sector hospitals are graded as to what type of care should be provided and resource allocation is determined by such grades, whether you are a district, regional or provincial, these grades should not compromise the care required by patients who are in dire need of a bed in rural regions. De Beer, Brysiewicz and Bhengu (2011) stated that lower level hospitals have fewer ICU beds than tertiary hospitals because tertiary hospitals provide advanced therapeutic interventions unavailable in lower level hospitals.

Giannini and Consonni (2006) also found that shortage of beds occurred every day in many ICUs. Robert et al. (2012) added that political and economic influences are major determinants of the allocation of ICU beds and that some patients might not be admitted to ICUs because, according to the admission criteria, they might be classified as being too ill or too well for ICU to be of help. Cheng et al. (2014) also mentioned that poor prognosis is often related to delayed ICU admission because of a shortage of ICU beds.

Breathing is vitally important for the human body. Infections, trauma and diseases can affect a person’s ability to breathe causing respiratory failure requiring mechanical ventilation to live. Participants indicated that patients shared monitors because of poor functioning of ventilators and the principle of continuous monitoring was not maintained. There was no proper maintenance and equipment were not serviced regularly. Krishnamoorthy, Vavilala and Mocke (2014) indicated that ventilators saved many lives in developed countries and that many patients died of reversible conditions in developing countries because of the lack of ventilators and poor patient management. Furthermore, the authors stated that ventilators could be vulnerable machines and require appropriate maintenance. The study by De Mello and Barbosa (2013) has shown that the equipment used in ICUs could cause harm to patients if they were not subjected to proper selection, acquisition processes and maintenance. In order to increase patients’ safety, adequate resources are required, and the availability of such machines in ICU is vital. Esteban et al. (2013) described the importance of having ventilators, which contributed to decreased mortality in mechanically ventilated patients.

Participants in this study indicated that they also experienced challenges because of insufficient pharmaceuticals and material resources. The ICU of a regional hospital lacked material resources and medicines that are standard in every ICU, and health care professionals had to frequently write motivations to acquire those resources. These findings concur with the findings of Bigdeli et al. (2015), indicating that some financing bodies contained medicine costs and prevented waste by limiting access to some medicines. However, this strategy compromised equity and quality of care. The study by Cameron et al. (2011) found that shortage of medicines in the public sector was related to poor maintenance of stock levels, the inability to forecast needs accurately, the inefficient purchasing and distribution systems or the leakage of medicines for private resale. Bigdeli, Peters and Wagner (2014) mentioned that spending money on medicines should be equitable in all population groups, which should be the case in rural poor settings. Provision of efficient and effective critical care requires that stock levels of day-to-day provision of care are adequate; however, participants had to overstock and hoard because of poor stock maintenance. The study of Heydari, Najar and Bakhshi (2015) on resource management among intensive care nurses validates the current study’s findings that equipment should be procured and properly handled as overstocking results in some stock expiring before use. The participants also reported that they had to use the same suction catheter for the whole day which compromised the patients to risk of infection. The presence of an artificial airway impairs the pulmonary defence mechanisms of patients in ICU, and patients are more at risk of invasion of microorganism creating a direct route for the bacteria to enter the lungs. Gillespie (2009) indicates that multidrug-resistant (MDR) strains of the pathogens are increasing in incidence both locally and internationally, and tuberculosis and HIV are co-morbid diseases that are most prevalent in Limpopo province. Chhugani and James (2017) narrated similar problems of day-to-day resources required for rendering nursing care which ranged from the inadequacy of life-saving supplies and equipment including drugs that are to be administered intravenously, adrenaline, oxygen and autoclaves to relatively cheap supplies including gauze and cotton wool. Shortages of supplies can compromise the quality of care.

Rural hospitals face human resource challenges as experienced doctors and specialists are not keen to work in rural settings. Doctors stay in rural areas only to complete community service and thereafter leave to seek employment in well-resourced settings in urban areas. Research findings indicate that nurses took decisions and patients were not reviewed on time as there was no specific doctor allocated in ICU. Lee et al. (2014) stated the advantages of the presence of intensivist in ICUs as the timely and frequent titration of therapy by being physically present in the ICU. Park et al. (2014) acknowledged that although there are shortages of ICU doctors, they are needed to staff ICUs. Wilcox et al. (2014) specified that according to the European Society of Intensive Care Medicine (ESICM) guidelines, ICUs require specialised ICU doctors preferably for 24 h per day.

The participants further revealed that there is no specific doctor allocated in the ICU; doctors in casualty are to see patients and usually come late in the afternoon. In their study, Juneja, Nasa and Singh (2012) found that daily rounds by an intensivist improved patients’ outcomes, reduced the patients’ duration of stay in the ICU and reduced hospital costs. Park et al. (2014) concurred that without an intensivist it would be difficult to maintain the quality of critical care and that patients will not always receive the appropriate treatment. Wallace et al. (2012) also supported the view that a 24-h doctor in ICUs would clarify orders and prevent medical errors.

Professional nurses also felt challenged when they had to perform tasks that were not within their scope of practice, and some doctors did not have experience of working in the ICU and needed assistance from the experienced ICU nurses, but at times conflict arose when doctors had to endorse some changes done by the ICU nurses in their absence. Wallace et al. (2012) suggest that the availability of a specialised doctor in an ICU results in the initiation of proper treatment as soon as the patient is admitted to the ICU, no delays occur during emergencies, patient care is continuous and constant advice is available 24 h per day.

Furthermore, the study findings indicate the shortage of ICU-trained registered nurses and the use of lower categories of nurses to care for ICU patients, which result in poor supervision on the provision of care provided. Matlakala and Botha (2016) reported that the increase in technical and patient complexity requires specific education to maintain and develop skills, with an increasing awareness that basic nursing education does not develop the requisite levels of specialised skills and knowledge to practise safely in critical care units. St-Pierre et al. (2011) also indicated that most experienced nurses in ICU are obliged to act as preceptors to nurses not trained in ICU and the time allotted to integrating new nurses into the ICU is often too short to allow them to appropriate the role of intensive care nurse, and ICU nurses are overwhelmed by the complexity of ICU that demands specific technical, technological and relational skills. St-Pierre et al. (2011) indicated that ICU nurses deal with nursing situations that are characterised by high levels of complexity, uncertainty, instability and variability resulting in stress.

Shortages of trained ICU nursing personnel contribute to negligence and provision of inadequate nursing care because of mistakes and omissions, the workload is too much for the trained staff and at times working night duty for prolonged periods can be stressful. The findings are confirmed by Ferrer et al. (2014), indicating that lack of trained staff results in increased workloads compromising patient care. Matlakala, Bezuidenhout and Botha (2014) also indicated that units should be staffed with trained and experienced nurses. Bhagwanjee and Scribante (2007) reiterated that for safe and effective intensive care practice there must be dedicated medical and nursing staff. Gjerberg et al. (2010) are also of the opinion that the lack of staff and skills causes poor end-of-life care. Standards for safe staffing in ICUs require that every patient in an ICU or critical care unit must have immediate access to a registered nurse with a post-registration ICU qualification (British Association of Critical Care Nurses 2010).

In health care settings, there are grey areas where nurses are able to perform tasks that are performed by the doctor, but a collegial relationship must occur in discussing the patients’ treatment with nurses. Intensive care unit nurses are analytic and independent practitioners in their own right when caring for critical patients. Participants faced challenges with doctors who did not want to endorse changes done by nurses such as changing IV fluids after blood results. St-Pierre et al. (2011) also maintained that ICU nurses love challenges, risk-taking and see stressful situations as motivating and challenging rather than a threat. St-Pierre et al. (2011) further alluded that nurses usually see emergencies, cases of cardiopulmonary arrest, patients who are unstable or in critical condition, responsibilities, decision-making, unfamiliar situations as well as the aspects of a situation they may be unacquainted with as stimulating or as challenging and these lead them to outdo themselves. Nurses are also often overburdened with other non-nursing duties, but continue to perform such tasks for the sake of patient care. Chhugani and James (2017) reported that in almost all health care settings, nurses undertake roles that are not part of their profession such as billing, record keeping, inventory, laundry, diet and physiotherapy, thereby diminishing time for patient care; hence, they are left with minimal time to carry out their actual roles and responsibilities.

## Limitations of the study

The study was conducted in one hospital with a purposive sample of 17 health care professionals. This limits the generalisability of the findings to a wider range of ICUs or even other hospitals in the rest of South Africa. The information provided by the interviewed doctors and nurses was accepted without conducting observations.

## Recommendations

- Government in the Limpopo province should improve the infrastructure in the regional hospitals and increase the bed capacity to ensure equitable access to critical care in relation to the demand of the district.
- Guidelines to be developed for bed occupancy or when the unit is full could be valuable, and a high care unit should be established for patients who do not need intensive care.
- Procurement processes should be managed properly in allocating high technology equipment such as ventilators and monitors to regional hospitals to ease the burden of unnecessary transfer of patients to provincial hospital for ventilation.
- High technology equipment should be serviced regularly for their durability and be replaced as required by the manufacturers’ specifications.
- An intensivist or experienced physician should be allocated to an ICU to manage emergency cases, instead of inexperienced doctors who are hesitant in making decisions.
- Intensive care unit must be staffed with specifically trained staff and nurses to be given study leave to increase skilled personnel. Patients are safer when trained staff care for them.
- Intensive care units must have all the pharmaceuticals and consumables at all times. Hospital policy on procurement of supplies must ensure that adequate levels of medicines and consumables are maintained at all times.
- Further research should be conducted on moral stress related to challenges experienced by health care professionals working in ICUs.

## Conclusions

The availability of material and human resources is essential for the proper management of critically ill patients. These shortages caused dangerous practices that were a risk for patients’ lives. The number of ICU beds was insufficient for the population served by ICU. No intensivist was specifically assigned to the ICU for implementing instant interventions in emergencies as the hospital lacks specialist doctors. Equipment such as mechanical ventilators, monitors and blood gas machines were not properly maintained and health care professionals were not frequently trained on how to use new pieces of equipment properly.
